# Longitudinal assessment of anchored transponder migration following lung stereotactic body radiation therapy

**DOI:** 10.1002/acm2.12454

**Published:** 2018-11-01

**Authors:** Andrew M. McDonald, Tyler Colvin, D. Hunter Boggs, Sharon A. Spencer, Richard A. Popple, Ravinder Clayton, Douglas Minnich, Michael C. Dobelbower

**Affiliations:** ^1^ Department of Radiation Oncology University of Alabama at Birmingham Birmingham AL USA; ^2^ University of Alabama at Birmingham School of Medicine Birmingham AL USA; ^3^ Grandview Medical Center Thoracic Surgery Birmingham AL USA

**Keywords:** electromagnetic transponder beacons, image guided radiation therapy, lung cancer, stereotactic body radiation therapy

## Abstract

**Purpose:**

To assess the long‐term stability of the anchored radiofrequency transponders and compare displacement rates with other commercially available lung fiducial markers. We also sought to describe late toxicity attributable to fiducial implantation or migration.

**Materials and methods:**

The transponder cohort was comprised of 17 patients at our institution who enrolled in a multisite prospective clinical trial and underwent bronchoscopic implantation of three anchored transponders into small (2–2.5 mm) airways. We generated a comparison cohort of 34 patients by selecting patients from our institutional lung SBRT database and matching 2:1 based on the lobe containing tumor and proximity to the bronchial tree. Assessment of migration was performed by rigidly registering the most recent follow‐up CT scan to the simulation scan, and assessing whether the relative geometry of the fiducial markers had changed by more than 5 mm. Toxicity outcomes of interest were hemoptysis and pneumothorax.

**Results:**

The median follow‐up of patients in the transponder cohort was 25.3 months and the median follow‐up in the comparison cohort was 21.7 months. When assessing the most recent CT, all fiducial markers were within 5 mm of their position at CT simulation in 11 (65%) patients in the transponder group as compared to 23 (68%) in the comparison group (*P* = 0.28). One case of hemoptysis was identified in the transponder cohort, and bronchoscopy confirmed bleeding from recurrent tumor; no cases of hemoptysis were noted in the comparison cohort. No case of pneumothorax was noted in either group.

**Conclusion:**

No significant difference in the rates of fiducial marker retention and migration were noted when comparing patients who had anchored transponders placed into small airways and a 2:1 matched cohort of patients who had other commercially available lung fiducial markers placed. In both groups, no late or chronic toxicity appeared to be related to the implanted fiducial markers.

## 1 INTRODUCTION

Stereotactic body radiation therapy (SBRT) has revolutionized the treatment of early stage non‐small cell lung cancer (NSCLC) for medically inoperable patients, and those who decline surgical resection. Since SBRT requires delivery of ablative radiation doses with steep dose gradients, accounting for target motion is critical. Early lung SBRT techniques required large internal target volume (ITV) expansions in order to account for target motion throughout the respiratory cycle.^1^ As SBRT utilization has evolved, a number of techniques have been developed to reduce the amount of normal lung tissue within the treatment volume. Respiratory gating is commonly utilized to restrict treatment delivery to only a portion of the respiratory cycle, typically near end‐expiration, and therefore reducing the necessary ITV margin.^2^ A potential concern with respiratory gating is that many gating systems rely on the position of an external marker, which has the potential to be a poor surrogate for target motion.^3^, ^4^, ^5^

To mitigate the concern that movement of the gating marker and tumor may be discordant, real time radiographic tracking of implanted fiducial markers can be combined with respiratory gating. The use of triggered planar imaging to assess fiducial marker position improves the accuracy of treatment delivery,^6^ but this technique has a number of limitations. For instance, the tumor is periodically assessed rather than continuously monitored, treatment delivery remains tied to the respiratory cycle rather than tumor position, and fiducial deviation is assessed visually and therefore subject to human error. In order to overcome these limitations, the Calypso^®^ System (Varian Medical Systems, Palo Alto, CA, USA) has been suggested as a potential solution.^7^

The Calypso System consists of electromagnetic transponders paired with a detector array. The Calypso System has been validated for providing real‐time tumor tracking within a variety of solid tissues.^8^ In order to extend this technology to lung tumor localization, a 5‐legged nitinol stabilization system has been developed to anchor the transponders within the lung tissue. The anchored transponders are placed within small airways, on the order of 2 mm diameter, via navigational bronchoscopy. The short‐term stability and accuracy of anchored transponders was recently the subject of a prospective clinical trial, but no long‐term stability and safety data have yet been reported. The purpose of this study was to assess the long‐term stability of anchored transponders. We also sought to describe late toxicity attributable to fiducial implantation or migration. Finally, we compared migration rates with other commercially available lung fiducial markers as a surrogate for the possibility of unexpected future clinical manifestations.

## 2 MATERIALS AND METHODS

### 2.A Patient cohorts

The transponder cohort consisted of all patients at our institution who underwent placement of anchored transponders into small airways as part of a prospective clinical trial (NCT01396551); no patients who underwent transponder placement were excluded. A comparison cohort was constructed by first reviewing the records of all patients who underwent lung SBRT at our institution between 2010 and 2016. For each transponder case, two matched patient cases were selected who had undergone placement of other types of radiopaque lung fiducial markers. Matching criteria were lung lobe and proximity to the proximal bronchial tree (peripheral vs central using the NRG Oncology definition^9^).

### 2.B Procedures and follow‐up protocol

Placement of markers was performed following a pathologic diagnosis of malignancy. In the transponder cohort, three anchored transponders were placed via navigational flexible bronchoscopy into small airways (2–2.5 mm) near the tumor. In the comparison cohort, 2–3 radiopaque fiducial markers were placed near the tumor either via bronchoscopy or via CT‐guided transthoracic needle. In all cases an immediate chest radiograph was performed to confirm retention of the fiducial markers.

4‐Dimension CT simulation for SBRT planning was typically performed within 2 weeks of fiducial marker placement. In most cases, peripheral tumors were prescribed a dose of 54 Gy in 3 fractions and central tumors were prescribed a dose of 48–52.5 Gy in 4–5 fractions. One patient received fractionated therapy (60 Gy in 30 fractions) due to mediastinal adenopathy that was identified at the time of simulation. Following treatment, a baseline chest CT was performed within 3 months and repeated every 6 months coinciding with clinical toxicity assessment. Additional thoracic imaging and bronchoscopy were performed only if clinically indicated.

### 2.C Assessment of fiducial movement

**Figure 1:**
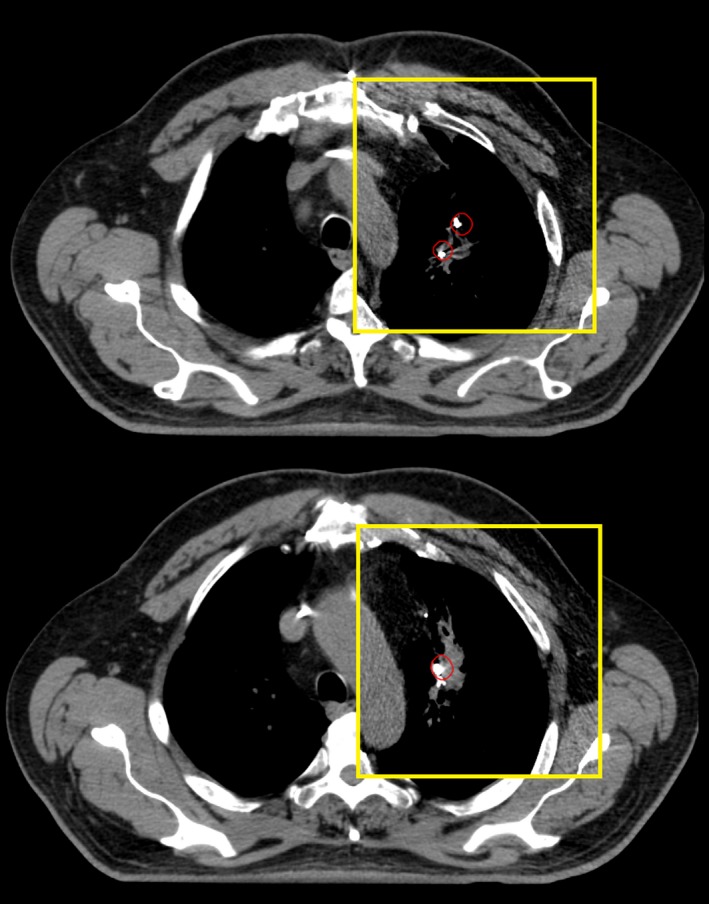
Example case of anchored transponders with stable positioning. Three anchored transponders were placed in the left upper lobe, two superior transponders in upper panel and one slightly inferior in lower panel. Follow‐up CT scan (yellow frame) at 14 months after treatment superimposed on CT simulation scan shows all three transponders retained their relative position within a 5 mm radius of their position at simulation (red circle).

**Figure 2:**
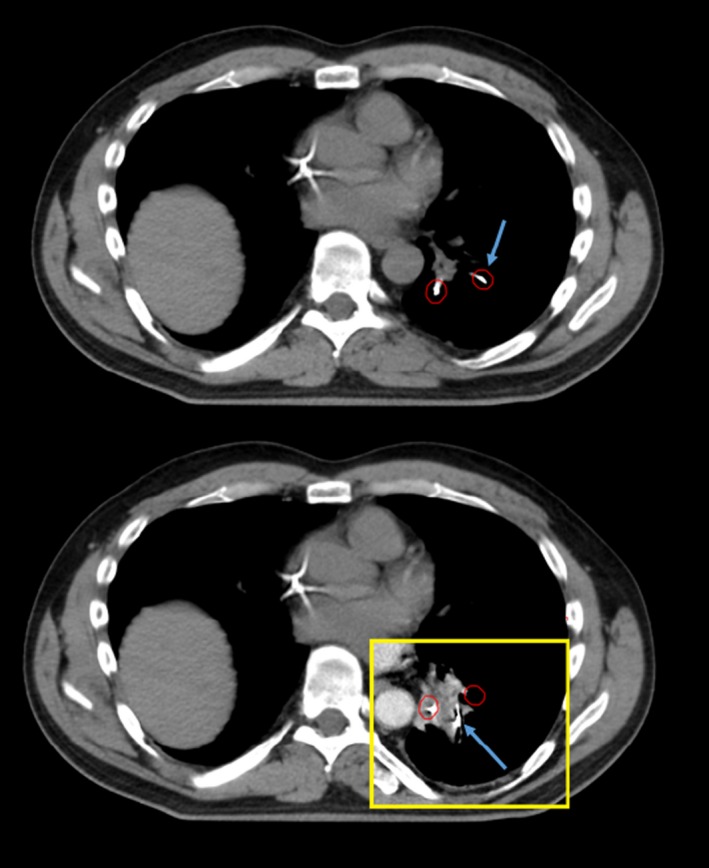
Example case of anchored transponder displacement due to evolution of postradiation fibrosis. Three anchored beacons were placed within the left lower lobe. Upper pane is an angled axial slice from CT simulation that shows position of 2 anchored beacons. Follow‐up CT scan (yellow frame, lower panel) at 24 months after treatment superimposed on CT simulation scan shows one beacon (blue arrow, both panes) has been displaced more than 5 mm from the initial position (red circle) but remains within the area of postradiation fibrosis

**Figure 3:**
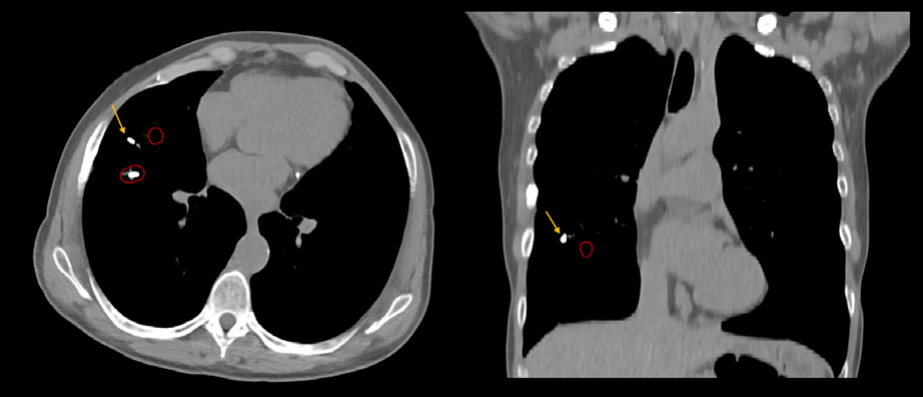
Only one instance of transponder displacement apart from post‐SBRT fibrotic change was noted. Three anchored transponders were placed within the right middle lobe. Representative angled axial (left pane) and angled coronal (right pane) slices are shown on the follow‐up CT scan, which was performed 19 months after SBRT was completed. The follow‐up CT was registered to the CT simulation scan and the red circle represents a 5 mm expansion around the initial position of the anchored transponder. One transponder was noted to have been displaced superiorly and laterally (yellow arrow). No symptoms were attributed to fiducial displacement at clinical follow‐up.

The simulation CT scan was considered as the reference image for baseline fiducial location. On the 4‐dimensional simulation CT data set, the implanted markers were manually segmented on the earliest phase of the respiratory cycle for which treatment was planned (corresponding to when the treatment gate opens) using Varian Eclipse software and a uniform 5 mm spherical expansion structure around each marker was generated (depicted in red in Figs. 1, 2, 3). The most recent CT scan, up to 2.5 yr post‐SBRT, was then imported for registration to the simulation CT scan. Scans beyond 2.5 yr in the comparison cohort were not considered since no patient within the transponder cohort had been followed longer than that period of time. The follow‐up CT scan was then rigidly registered to the simulation scan (Fig. 1). Registration methods consisted of a rigid 3‐point registration to the fiducial markers with manual adjustments allowed. Unreasonable distortion of patient anatomy (eg, 90 degree rotation) was not allowed. Fiducial displacement was said to have occurred if the follow‐up CT could not be registered in such a way that all markers were within the 5 mm sphere around the initial position. The 5 mm threshold to define displacement was based on the investigators’ judgment regarding the magnitude of fiducial position changes that were not likely due to respiratory motion or CT acquisition technique.

For cases where marker displacement was noted, the follow‐up CT scan was further assessed to determine whether the displaced marker(s) was located within an area of fibrosis or normal appearing lung parenchyma. In each case, whether marker displacement was most likely due to evolving postradiation fibrosis (Fig. 2) or to migration (Fig. 3) was recorded.

### 2.D Analysis and regulatory review

All statistical analyses were performed using SPSS Statistics 24 (IBM Corporation, Armonk, New York, USA). Differences in frequencies between the two patient cohorts were assessed using the *χ* ^2^ test. An unconditional test of frequencies was chosen because of the large number of potential pairings between patients in the transponder and comparison cohorts.^10^ This study was reviewed and approved by the Institutional Review Board at the University of Alabama at Birmingham. This research was funded by Varian Medical Systems. The funding agent had no role in the study design, data collection, analysis, or results interpretation.

## 3 RESULTS

### 3.A Baseline characteristics

A description of baseline patient and treatment characteristics is given in Table 1. Markers placed in the comparison cohort were gold seeds with nitinol coils in 18 (53%) patients, fibered platinum vascular occlusion coils in 12 (35%) patients, and were of unspecified type in 4 (12%) patients. In the transponder group all transponders (51 transponders in 17 patients) placed at bronchoscopy were accounted for on the simulation scan. In the comparison group four patients had only one fiducial marker visible, though in each case two were documented to have been placed. The median delay from marker placement until CT simulation was 4 days (Range: 3–19 days) in the transponder group and 9 days (Range: 1–48 days) in the comparison group.

**Table 1:** Baseline characteristics

		Anchored transponder cohort	Radiopaque fiducial cohort
		# of patients, N (%)	
Lobe with implanted fiducial	RUL	5 (29)	10 (29)
	RML	2 (12)	4 (12)
	RLL	1 (6)	2 (6)
	LUL	6^a^ (35)	12 (35)
	Lingula	0	0
	LLL	3 (18)	6 (18)
Central?	Yes	8 (47)	14 (41)
	No	9 (53)	20 (59)
Prescription dose	54 Gy/3 fractions	5 (29)	14 (41)
	52.5 Gy/5 fractions	10 (59)	12 (35)
	Other	2 (12)	8 (24)
Number of fiducials identified at simulation	3	17 (100)	8 (24)
	2	0	22 (65)
	1	0	4 (12)
Type of fiducial	Anchored transponders	17 (100)	0
	Anchored gold seeds	0	18 (53)
	Vascular coils	0	12 (35)
	Undocumented	0	4 (12)
Days fiducial placement until CT simulation	Median (range)	4 (3–19) days	9 (1–48) days

a One patient with two transponders placed in LUL and one in LLL was treated as LUL for purposes of case matching.

### 3.B Transponder position over time

The median follow‐up of patients in the transponder cohort was 25.3 months and the median follow‐up in the comparison cohort was 21.7 months. Across both groups, no fiducial marker noted at the time of CT simulation was unaccounted for at the most recent CT. When assessing the most recent CT, all markers were within 5 mm of the position on the CT simulation scan in 11 (65%) patients in the transponder group as compared to 23 (68%) in the comparison group (*P* = 0.28). Of the six patients with marker displacement within the transponder group, five instances appeared to be related to radiation fibrosis around one or more transponders. Of the 11 patients with fiducial displacement within the comparison group, four instances of fiducial displacement appeared to be related to progressive radiation fibrosis.

### 3.C Toxicity

No patient within either group developed a pneumothorax at any time point. One patient in the transponder group experienced gross hemoptysis approximately 1 yr from fiducial implantation, but bronchoscopic evaluation confirmed the bleeding was due to local tumor recurrence. No patient in the comparison group experienced gross hemoptysis.

## 4 DISCUSSION

The Calypso System utilizing electromagnetic transponders has multiple characteristics that offer potential advantages over current respiratory motion management techniques for lung SBRT. In contrast with traditional fiducial markers combined with respiratory gating, electromagnetic transponders provide nearly constant target localization, with their position updated approximately 25 times per second.^11^ Decreased target position uncertainty should allow for a reduction in ITV and PTV margins and therefore reduce the amount of nontumor tissue within the treatment volume. When combined with the Dynamic Edge Gating system, the Calypso System obviates the need for an external surrogate gating marker since a beam‐hold is imposed if the transponders move beyond a prespecified margin. Gating treatment delivery based on target position rather than the respiratory cycle may also improve the overall efficiency of treatment, particularly for patients with irregular respirations, by reducing unnecessary beam‐holds.

Anchored transponders are placed via bronchoscopy and utilize a 5‐legged nitinol stabilization system to aid retention.^12^ Preliminary evidence supports the short‐term stability and accuracy of the anchored transponders.^13^ Fixation of the transponders for up to 60 days was similar to that of spherical gold markers in a canine model,^14^ but longer follow‐up in human subjects has not yet been assessed. Since the long‐term behavior of anchored transponders has not yet been described, clinicians may be reticent to adopt this promising new technology. This study was therefore undertaken to investigate the long‐term movement of anchored transponders placed within the lung and to describe any unexpected late morbidity associated with transponder placement. All the implanted anchored transponders remained at the time of CT simulation. With a median follow‐up of 25.7 months, the relative position of three implanted anchored transponders was maintained within 5 mm in two‐thirds of cases (including the patient case where conventional fractionation was utilized). Little data are available regarding the long‐term behavior of implanted fiducial markers within the lung. Therefore, to provide context for the longitudinal assessment of the anchored transponders, we also assessed a cohort of patients with similar tumor characteristics who had undergone placement of radiopaque fiducial markers. The rate of long‐term fiducial displacement was 32% with a median CT follow‐up of 21.7 months. The fact that all fiducials were accounted for at most recent CT is likely a corollary of the fact that in this study the baseline fiducial assessment was at the time of simulation rather than initial implantation.

In the post‐SBRT setting, displacement of implanted fiducial markers from their initial position may be due to migration of the markers through lung or due to changes in the surrounding anatomy due to the evolution of post‐radiation fibrosis. Fibrosis following SBRT follows many patterns^15^, ^16^ and retraction of the treated area toward the mediastinum is common. For the majority of cases of fiducial displacement across both groups in this study, the tissue surrounding displaced markers had characteristics of post‐SBRT fibrosis. Five of the six (83%) patient cases where transponder displacement was noted, the displaced transponders were within an area of progressive fibrosis at the SBRT treatment site. This supports a hypothesis that true migration due to fiducial instability may only account for a minority of cases of overall fiducial displacement. The one displaced transponder in the absence of postradiation fibrosis remained within the same lobe of the lung and did not result in any clinically apparent toxicity.

Rates of acute pneumothorax exceeding 20% were initially described in early series that utilized trans‐thoracic fiducial placement techniques,^17^, ^18^ but this has become rare in the era of bronchoscopic placement. The fact that no patient in this series developed a pneumothorax is consistent with recent reports.^19^ Rare complications from fiducial marker migration into central mediastinal structures have been reported,^20^, ^21^ but none were observed in this cohort of 17 patients with anchored transponders, and 34 patients with other lung fiducial markers. Since the anchored transponders are placed within small airways, we assessed for clinical manifestations of local reaction and/or erosion such as hemoptysis. Only one case of hemoptysis was recorded after SBRT in the group and bronchoscopic evaluation identified the cause of bleeding as recurrent tumor.

The primary limitations of this study are its retrospective nature and modest sample size. To reduce the likelihood of selection bias, no patient who underwent transponder placement was excluded from this analysis. The purpose of constructing a matched comparison group was to prevent an imbalance of fiducial marker anatomic implantation sites between the groups. We recognize the possibility of inadvertently introducing bias as an intrinsic limitation of matched cohort studies; therefore, we used a 2:1 match in order to minimize this possibility. The single institution nature of this study must also be taken into account when interpreting these data. The University of Alabama at Birmingham is a high volume lung SBRT center with more than 10 yr of experience with bronchoscopic placement of fiducial markers.

In summary, no difference in the rates of fiducial marker migration were appreciated when comparing patients who had anchored transponders placed into small airways and a cohort of patients who had other commercially available lung fiducial markers placed. In both groups, all of the fiducial markers identified at the time of CT simulation were accounted for at most recent follow‐up. No patient experienced a late complication that was attributable to the beacons or fiducial markers. The great majority of positional shifts greater than 5 mm over time for the anchored transponders appear to be related to post‐radiation fibrosis. These data support the conclusion that the long term clinical behavior of implanted anchored transponders is similar to that of other lung fiducial markers.

## FUNDING

This research was funded by Varian Medical Systems. The funding agent had no role in the study design, data collection, analysis, or results interpretation.

## CONFLICTS OF INTEREST

AM, DB, RP, and MD receive (or have received) research funding from Varian Medical Systems.
